# Effects of antiepileptic drugs on hippocampal neurons coupled to micro-electrode arrays

**DOI:** 10.3389/fneng.2013.00010

**Published:** 2013-11-19

**Authors:** Ilaria Colombi, Sameehan Mahajani, Monica Frega, Laura Gasparini, Michela Chiappalone

**Affiliations:** ^1^Department of Neuroscience and Brain Technologies, Istituto Italiano di TecnologiaGenova, Italy; ^2^Department of Informatics, Bioengineering, Robotics and System Engineering, University of GenovaGenova, Italy

**Keywords:** valproate, carbamazepine, bicuculline, *in vitro* culture, bursts, spikes

## Abstract

Hippocampal networks exhibit spontaneous electrophysiological activity that can be modulated by pharmacological manipulation and can be monitored over time using Micro-Electrode Arrays (MEAs), devices composed by a glass substrate and metal electrodes. The typical mode of activity of these dissociated cultures is the network-wide bursting pattern, which, if properly chemically modulated, can recall the ictal events of the epileptic phenotypes and is well-suited to study the effects of antiepileptic compounds. In this paper, we analyzed the changes induced by Carbamazepine (CBZ) and Valproate (VPA) on mature networks of hippocampal neurons in “control” condition (i.e., in the culturing medium) and upon treatment with the pro-convulsant bicuculline (BIC). We found that, in both control and BIC—treated networks, high doses (100 μM–1 mM) of CBZ almost completely suppressed the spiking and bursting activity of hippocampal neurons. On the contrary, VPA never completely abolish the electrophysiological activity in both experimental designs. Interestingly, VPA cultures pre-treated with BIC showed dual effects. In fact, in some cultures, at low VPA concentrations (100 nM–1 μM), we observed decreased firing/bursting levels, which returned to values comparable to BIC-evoked activity at high VPA concentrations (100 μM–1 mM). In other cultures, VPA reduced BIC-evoked activity in a concentration-independent manner. In conclusion, our study demonstrates that MEA-coupled hippocampal networks are responsive to chemical manipulations and, upon proper pharmacological modulation, might provide model systems to detect acute pharmacological effects of antiepileptic drugs.

## Introduction

In recent years the need of efficient neuropharmacological and neurotoxicological testing *in vitro* is increasing, as there are new directives to restrict animal use for laboratory tests (Johnstone et al., 2010). New experimental strategies based on alternative methods, in which the use of time, materials, and animals is reduced and refined or animal use is completely replaced, are required. Thus, *in vitro* assessment of neurophysiological function could be used to screen chemicals for potential neuroactive or neurotoxic effects (Defranchi et al., 2011).

To date, one of the most promising tools for neuropharmacological tests is the Micro-Electrode Array (MEA). Developed at the beginning of the'80 (Gross et al., 1977; Pine, 1980), MEA technology has been recognized as a standard experimental approach for *in vitro* long-term electrophysiological and neuropharmacological investigations (Gross et al., 1997; Gramowski et al., 2004). Primary neurons from rodents cultured over MEAs remain spontaneously active and stable for several months (Gross et al., 1982; Potter and DeMarse, 2001; Gramowski et al., 2004). Moreover, cultured neuronal networks respond to neurotransmitters and their blockers in a similar way as the *in vivo* situation (Streit, 1993; Gramowski et al., 2000; Martinoia et al., 2005), providing an excellent tool to study how pharmacological compounds can influence the electrophysiological behavior (Gross et al., 1997; Morefield et al., 2000; Keefer et al., 2001; Xia and Gross, 2003; Parviz and Gross, 2007).

One of the major modes of activity of highly-connected cultures of dissociated neurons is globally synchronized bursting. Unlike *in vivo*, neuronal ensembles in culture maintain activity patterns dominated by global bursts for the lifetime of the culture (Wagenaar et al., 2005). *In vivo*, bursting occurs during development and plays a role in establishing appropriate connections (Ben-Ari, 2001). However, this phase lasts only for days or, at most, weeks. The persistence into maturity of bursting in culture may then be interpreted as a sign of developmental arrest (Corner et al., 2002). Moreover, this pattern of activity, if properly chemically modulated, can model the induction of epileptic activity (Furshpan and Potter, 1989; Vedunova et al., 2013). For this reason, this experimental preparation could be suited to study the effects on compounds which could abolish or reduce the incidence of epileptic “ictal” events.

Antiepileptic drugs are the mainstay of epilepsy treatment and act to suppress seizure severity and frequency. However, such drugs can also have paradoxical effects, exacerbating seizures in some patients. Carbamazepine (CBZ) is the drug of choice for the treatment of focal seizures; however, it commonly aggravates several generalized seizures types, including absence seizures. Sodium valproate (VPA) is widely used as antiepileptic drug with a broad spectrum of anticonvulsant activity and particular efficacy in generalized epilepsies (Dichter and Brodie, 1996). The mechanism of action of antiepileptic drugs, and specifically CBZ and VPA, has been investigated at the molecular and cellular levels. CBZ appears to act through the use-dependent blockade of voltage-gated sodium channels, hence inhibiting rapid burst firing neurons, while allowing normal non-bursting neuronal transmission (Kohling, 2002). The anticonvulsant action of VPA is generally ascribed to an enhancement of inhibitory neurotransmission mediated by GABA receptors (Rowley et al., 1995; Loscher, 1999), which increases hippocampal GABA levels (Biggs et al., 1992; Rowley et al., 1995) and inhibitory post-synaptic potentials (Preisendorfer et al., 1987). VPA also acts on excitatory neurotransmission by suppressing the responses mediated by NMDA receptors (Gean et al., 1994), blocks sodium ion channels and reduces T-type calcium currents (Kelly et al., 1990). However, how these mechanisms integrate at the neuronal network level to achieve the overall pharmacological effects remains unexplored.

In this work, we used hippocampal cultures coupled to MEAs to investigate the acute effects of increasing concentrations of antiepileptic drugs (i.e., VPA and CBZ) on neuronal network activity. We also studied the changes of the electrophysiological activity when the neural network was previously treated with bicuculline (BIC), in order to model an epileptic phenotype (Khalilov et al., 1997). To this aim, the electrophysiological activity of 101 networks was recorded and analyzed.

## Materials and methods

### Cell culture

Dissociated neuronal cultures were prepared from hippocampi of 18-day old embryonic rats (pregnant Sprague-Dawley female rats were obtained from Charles River Laboratories). All experimental procedures and animal care were conducted in conformity with institutional guidelines, in accordance with the European legislation (European Communities Directive of 24 November 1986, 86/609/EEC) and with the NIH Guide for the Care and Use of Laboratory Animals. Culture preparation was performed as previously described (Frega et al., 2012). Briefly, the hippocampi of 4–5 embryos were dissected out from the brain and dissociated first by enzymatic digestion in trypsin solution 0.125% (25–30 min at 37°C) and subsequently by mechanical dissociation with a fire-polished pipette. The resulting tissue was resuspended in Neurobasal medium supplemented with 2% B-27, 1% Glutamax-I, 1% Pen-Strep solution and 10% Fetal Bovine Serum (Invitrogen, Carlsbad, CA), at the final concentration of 36–40.000 cells/ml. Cells were plated onto 60-channel 6-wells MEAs previously coated with poly-D-lysine and laminin to promote cell adhesion (final density around 1200 cells/mm^2^) and maintained with 600 μl/well of nutrient medium (i.e., serum-free Neurobasal medium supplemented with B27 and Glutamax-I). They were then placed in a humidified incubator having an atmosphere of 5% CO_2_−95% air at 37°C. Half of the medium was changed weekly.

### Micro-electrode array recordings

**Figure 1:**
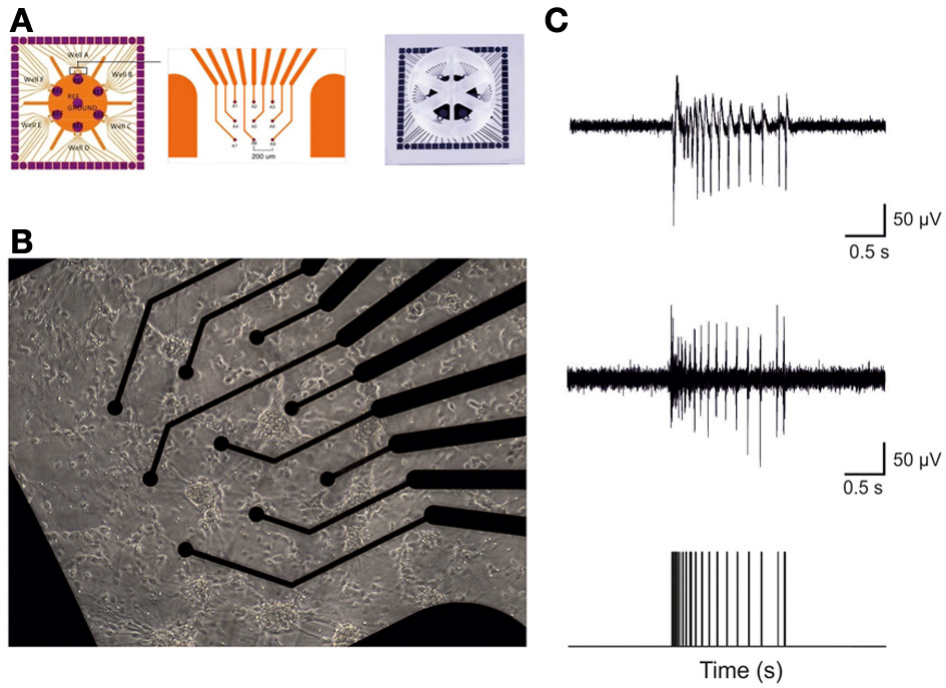
**Hippocampal cultures plated over 6-wells MEA devices form high-dense active networks. (A)** A 6-wells MEA device is characterized by six independent culture chambers, divided by a makrolon separator. Inside each well, nine electrodes and one big internal reference electrode allow recording of electrophysiological activity from a culture of dissociated neurons. **(B)** A 7DIV old culture of hippocampal neurons on top of 9 electrodes in a single MEA well. **(C)** Sample trace recorded from a single micro-electrode. The top panel reports a typical raw hippocampal burst. The middle panel depicts the same burst shown in the top upon high pass filtering at 300 Hz, to selectively analyze only Multi-Unit-Activity signals. The bottom panel exhibits the result of the spike detection procedure: only the timing of the spikes contains information and it is used for further analysis.

Microelectrode arrays (Multichannel Systems, MCS, Reutlingen, Germany) consisted of 60 TiN/SiN planar round electrodes (30 μm diameter; 200 μm center-to-center inter-electrode distance) divided into 6 separated wells (Figure 1). Each well was characterized by 9 recording electrodes, arranged in a 3 × 3 square grid, and one big ground electrode. The activity of all cultures was recorded by means of the MEA60 System (MCS). After 1200x amplification, signals were sampled at 10 kHz and acquired through the data acquisition card and MC-Rack software (MCS). To reduce thermal stress of the cells during the experiment, MEAs were kept at 37°C by means of a controlled thermostat (MCS) and covered by a PDMS cap to avoid evaporation and prevent changes in osmolarity (Blau et al., 2009). Additionally, we have settled a custom chamber to maintain a controlled atmosphere (i.e., gas flow of 5% CO_2_ and 95% O_2_ + N_2_) during the entire recording time, as reported in a previous paper (Novellino et al., 2011).

### Experimental protocol

The general protocol adopted for chemical stimulation included 60 min of recording in culture solution (Neurobasal + 2% B27 + 1% Glutamax-I 200 μM + 1% Penicillin-Streptomycin sol.), defined as a control condition. BIC (CAS number 485-49-4; Tocris, Bristol, UK) and VPA stock solutions were prepared and diluted in standard medium. The CBZ stock solution (100 mM) was prepared in a mixture of DMSO:water (50:50 volumes) and subsequently diluted in medium. The final concentration of DMSO was 0.5% for the highest concentration of CBZ used, and 0.05% for all the other concentrations. DMSO did not significantly alter the activity of hippocampal neuron networks. In fact, we tested the effect of 0.05–0.5% DMSO on a subset of hippocampal cultures (*N* = 7). We found that neither the low (i.e., 0.05%) nor the high (i.e., 0.5%) concentration of DMSO significantly changed the firing rate of hippocampal neurons (105.05 ± 14.64% for 0.05% DMSO; 94.38 ± 8.71 for 0.5% DMSO, [mean ± SE], *p* > 0.05, One-Way ANOVA). Firing rate absolute values were 3.21 ± 0.91 spikes/s in basal condition, 3.45 ± 0.89 spikes/s at 0.05% DMSO, and 2.61 ± 1.1 spikes/s at 0.5% DMSO (mean ± SE, *p* > 0.05, One-Way ANOVA). Bursting Rate (131.73 ± 21.07% at 0.05% DMSO and 89.74 ± 18.43% at 0.5% DMSO, [mean ± SE]), Burst Duration (103.67 ± 9.8% at 0.05% DMSO and 99.25 ± 15.2% at 0.5% DMSO, [mean ± SE) and Mean Frequency intra Burst (95.5 ± 3.0% at 0.05% DMSO and 87.5 ± 4.6% at 0.5% DMSO, [mean ± SE]) at different DMSO concentrations were not statistically different (*p* > 0.05, One-Way ANOVA) from control values, indicating that the vehicle did not significantly affected neuronal network activity, as also reported in other studies (Defranchi et al., 2011).

To evaluate the effects of VPA and CBZ on the spontaneous network activity, increasing concentrations (100 nM–1 mM) of the drugs were sequentially applied to the cultures by directly pipetting in the medium. For each concentration, the electrophysiological activity was recorded for 1 h. Since we noticed that mechanical perturbation due to the pipette injection in the medium could cause a temporary instability of the firing rate, we discarded the first 10 min of each recording phase. According to this technique, the presented data refer to a recording period of 50 min for each experimental phase. The total number of experiments (i.e., number of recorded wells) performed by following this protocol was: 12 for CBZ (24.2 ± 1.5 DIV) and 8 for VPA (23.5 ± 1.5 DIV). The two groups were not statistically different in terms of age of the tested cultures (*p* > 0.05, Student's *t*-test).

We also evaluated the effects of VPA and CBZ in cultures treated with BIC, a GABA_A_ receptor antagonist that acts as pro-convulsant of the central nervous system (Khalilov et al., 1997), to mimic *in vitro* the activity achieved by seizures. The activity induced by BIC was recorded for 1 h before adding increasing concentrations of CBZ or VPA. BIC was present throughout the experiment with CBZ and VPA. The total number of experiments (i.e., number of recorded wells) performed by following this protocol was: 37 for CBZ (20.0 ± 0.63 DIV) and 44 for VPA (20.8 ± 0.61 DIV). *In vitro* age (DIVs) was not statistically different in the two groups (*p* > 0.05, Student's *t*-test).

### Cell viability assay

Cell viability was assessed as described previously (Gasparini et al., 2004). Briefly, primary mouse hippocampal neurons were seeded on 96-well plates at 7^*^10^3^ cells/well and 18 days later incubated for 1 or 5 h in the presence of increasing concentrations of CBZ, VPA and/or the respective vehicles. In a subset of cultures, to examine any effect of mechanical or osmotic stress, vehicle (0.5% DMSO) or increasing concentrations of CBZ or VPA were sequentially added to the same wells using the same volumes and time schedule used for MEA experiments. After incubation, the media were replaced with serum free medium containing 0.5 mg/mL MTT [3-(4,5-dimethylthiazol-2-yl)-2,5-diphenyltetrazolium bromide] and the cells were further incubated at 37.C for 90 min. The medium was discarded and the crystals of violet formazan were dissolved in acidic isopropanol (isopropanol/1 M HCl 1/4 24: 1 v/v) by shaking at room temperatures for 30 min. Absorbance was read at 595 nm. Cell viability after drug treatments was expressed as percentage of vitality of untreated cells.

### Data analysis and statistics

Data analysis was performed off-line by using a custom software developed in MATLAB^©^ (The Mathworks, Natick, MA, USA) named SPYCODE (Bologna et al., 2010), which collects a series of tools for processing multi-channel neural recordings. The different steps of the analysis are briefly reported in the following.

#### Data filtering

Since hippocampal neurons tend to cluster more than cortical ones (data not shown), we often noticed a clear low-frequency component in the recorded signal. For this reason, we operated an off-line data filtering by using a high-pass Butterworth filter with cut-off frequency at 300 Hz in order to select only the Multi-Unit-Activity components of the signal, as reported in the literature (Quian Quiroga and Panzeri, 2009; Buzsáki et al., 2012).

#### Spike detection

A custom spike detection algorithm was used to discriminate spike events and to isolate them from noise (Maccione et al., 2009). Briefly, the algorithm is based on the use of three parameters: (1) a differential threshold (DT) set independently for each channel and computed as 6 or 7-fold the standard deviation (SD) of the noise of the signal; (2) a peak lifetime period (PLP) set to 2 ms; (3) a refractory period set to 1 ms. The algorithm scans the raw data to discriminate the relative minimum or maximum points. Once a relative minimum point is found, the nearest maximum point is searched within the following PLP window (or vice versa). If the difference between the two points is larger than DT, a spike is identified and its timestamp saved. The result of the spike detection procedure is a “point process,” i.e., a sequence of time stamps each of them indicating the occurrence of a spike.

#### Burst detection

Hippocampal networks show both random spiking activity and, in large majority, bursting behavior (Brewer et al., 2009; Leondopulos et al., 2012). A burst consists in a fast sequence of spikes, usually occurring simultaneously at many channels. Bursts consist of packages of spikes distributed over a range of a few milliseconds which generally last from hundreds of milliseconds up to seconds, and are separated by long quiescent periods. A custom burst detection method, whose input parameters were directly estimated from the inter-spike interval distribution of each channel, was used. The method exploited the logarithmic Inter Spike Interval Histogram (logISI) to extract the parameters needed for the analysis of each recording channel (Selinger et al., 2007). Specifically, the identification of the two principal peaks in the logISI and the minimum value between them provided a simple way to define the ISI threshold (ISIth) that best separated intra-burst ISI from inter-burst ISI and, hence, to detect bursts. Details can be found in a recent paper from our group (Pasquale et al., 2010).

Once spike and burst detection procedures were performed, we extracted several parameters describing the electrophysiological patterns, such as firing rate [spikes/s], bursting rate [bursts/min], burst duration [ms] and frequency intra bursts [spikes/s].

#### Drug-response curve

In order to derive the drug-response curves, we used two different thresholds: (1) the first was used to discriminate between active and inactive networks [minimum network firing rate = 0.01 spikes/s], (2) the second was used to discriminate between active and inactive channels in a single network [minimum single channel firing rate = 0.001 spikes/s]. It is worth underlying that the number of “active” (i.e., above the threshold) channels was calculated phase by phase and reported for each experimental group in the Results section. The parameters extracted through the spike and burst detection procedure (i.e., firing rate, bursting rate, burst duration, frequency intra burst) were normalized for each experiment (i.e., each MEA well) with regard to the corresponding values of the reference (control or BIC, depending on the performed experiment) activity for direct comparability. The same procedures have been described in a previous paper from our group (Frega et al., 2012).

#### Statistics

Data within the text are expressed as mean ± standard error of the mean (SE), if not differently specified. Statistical tests were employed to assess the significant difference among different experimental conditions. The normal distribution of experimental data was assessed using the Kolmogorov–Smirnov normality test. We then performed the One-Way ANOVA statistical test. When ANOVA gave a significant (*p* < 0.05) result, the *post-hoc* Bonferroni test was employed to assess differences between the initial condition (either the spontaneous or the BIC one, depending on the adopted experimental protocol) and the different concentrations of the added antiepileptic drug. Statistical analysis was carried out by using OriginPro (OriginLab Corporation, Northampton, MA, USA) and Sigma Stat (Systat Software Inc., San Jose, CA, USA).

## Results

We studied the acute effects of increasing concentrations of CBZ (*N* = 12 cultures) and VPA (*N* = 8 cultures) on the spontaneous activity of mature hippocampal neurons cultured onto 6-wells MEA (Figure 1).

**Figure 2:**
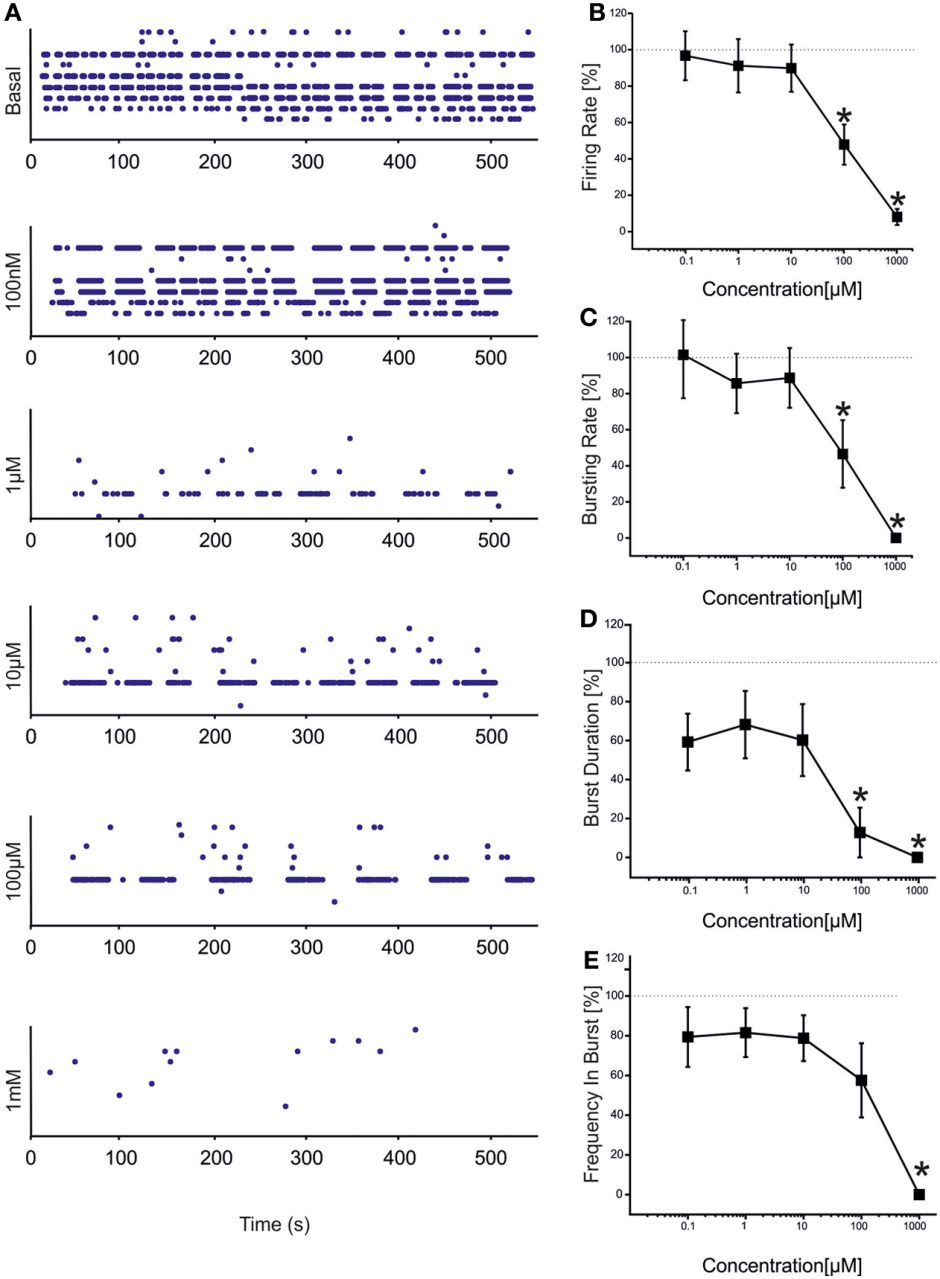
**Effects of increasing concentrations of CBZ on the firing and bursting dynamics of hippocampal networks. (A)** CBZ was added to the culture medium at increasing doses (100 nM, 1 μ M, 10 μ M, 100 μ M, 1 mM). The effects on the network activity were visible comparing the raster plots (500 s of activity acquired from a representative hippocampal culture) relative to the different experimental phases (each small dot represents a spike, each row an electrode; the 9 electrodes of a single well are reported). **(B)** In the analyzed group of experiments (*N* = 12; culture age 24.2 ± 1.5 DIV; firing rate in the basal condition 1.80 ± 0.51 spikes/s, mean ± SE), the firing rate upon drug administration significantly decreased compared to the control condition (dotted line at 100%) at 100 μM −1 mM. **(C)** The bursting rate followed a profile very similar to that observed for the firing rate, showing a significant decrease at 100 μM and 1 mM. **(D)** Burst duration started to statistically decrease at 100 μ M. **(E)** Mean frequency of spikes within the bursts statistically decreased at 1 mM. Statistics in **(B–E)** were performed using One-Way ANOVA followed by Bonferroni's test, ^*^p < 0.05.

CBZ induced a decrease in the number of spikes and bursts produced by the hippocampal networks (Figure 2), with a statistically significant decrease in both firing and bursting rates at high concentrations of the drug (100 μM and 1 mM; Figures 2A–E). At 1 mM, in particular, no burst was generated, indicating that the concentration was able to abolish the bursting activity. At lower concentrations (100 nM, 1 μM, 10 μM), on the contrary, bursting activity was present but the number of active channels was slightly less than in the other phases (100% in the control and at 100 nM; 95.77% at 1 μM, 10 μM, and 100 μM; 61.11% at 1 mM), as also shown in the raster plot of a representative experiment (Figure 2A).

**Figure 3:**
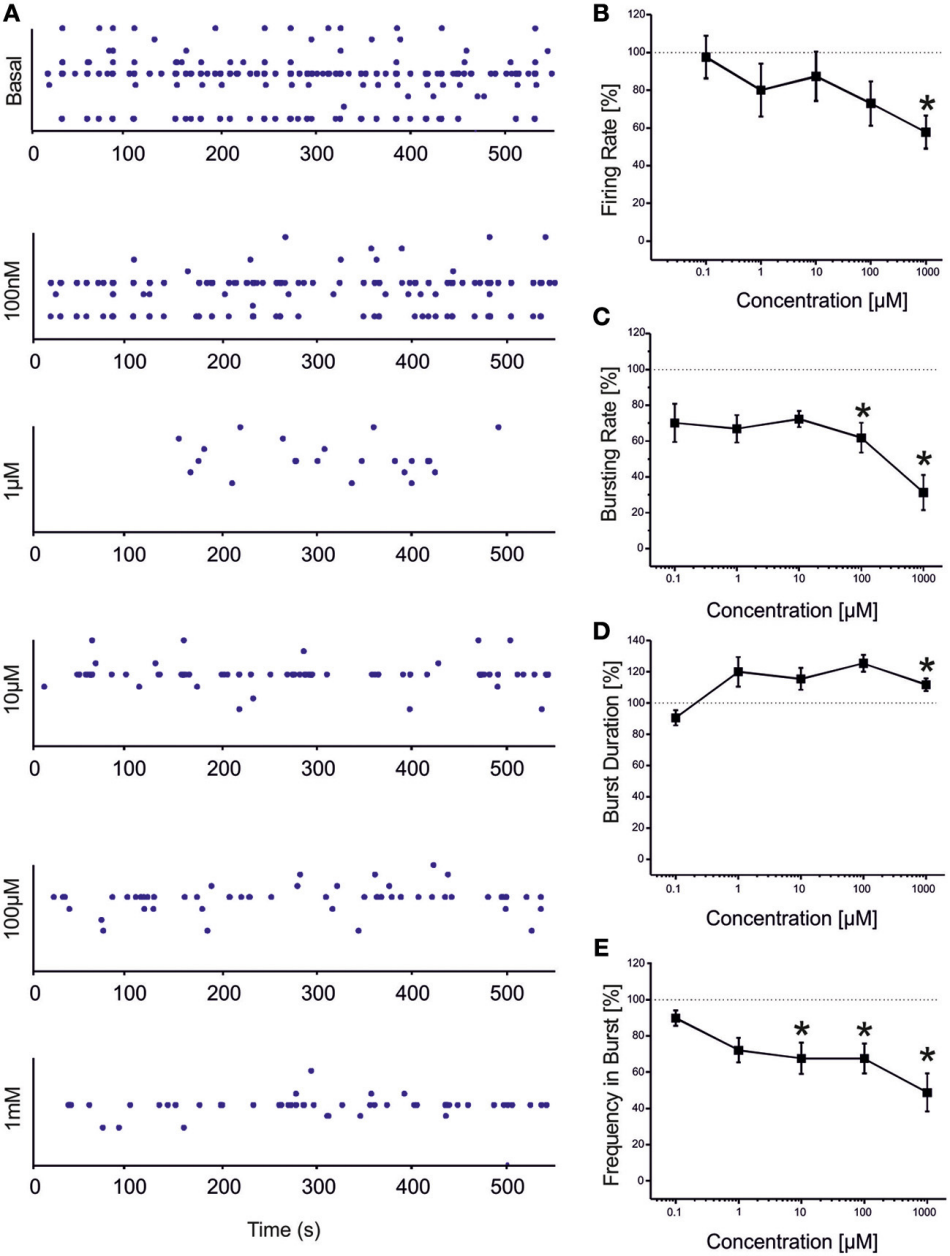
**Effects of increasing concentrations of VPA on the firing and bursting dynamics of hippocampal networks. (A)** VPA was added to the culture medium at increasing concentrations (100 nM, 1 μ M, 10 μ M, 100 μ M, 1 mM). The effects on the network activity were visible comparing the raster plots (500 s of activity acquired from a representative hippocampal culture) relative to the different experimental phases (each small dot represents a spike, each row an electrode; the 9 electrodes of a single well are reported). **(B)** We found that in the analyzed group of experiments (*N* = 8; culture age 23.5 ± 1.5 DIV, MFR 3.7 ± 1.1 spikes/s, mean ± SE), the firing rate significantly decreased with respect to the “spontaneous” (control) condition (dotted line at 100%) at VPA 1 mM. **(C)** The bursting rate significantly decreased at 100 μM −1 mM. **(D)** Burst duration showed a significant increase compared to the basal at VPA 1 mM. **(E)** Mean frequency of spikes within bursts statistically decreased starting from VPA 10 μM till 1 mM. Statistics in **(B–E)** were performed using One-Way ANOVA followed by Bonferroni's test, ^*^p < 0.05.

VPA showed a similar (but not equal) trend to that found for CBZ (Figure 3). Statistical analysis performed on the drug-response curves computed from different activity parameters (i.e., firing rate, bursting rate, burst duration and frequency intra burst, reported in Figures 3B–E) indicated that the decrease was significant at high concentrations of the drug (i.e., 100 μM–1 mM). However, unlike CBZ, both firing and bursting were never abolished by high concentrations of VPA. For concentrations ranging from 100 nM to 1 μM changes with respect to the basal condition were overall negligible. While a decrease in the rate of the events was appreciated at 100 μM and 1 mM, an increase in the duration of the bursting patterns was observed at 1 mM, indicating that a reduced number of bursts were generated, bursts lasted longer but had fewer spikes than in the absence of VPA. The percentage of active channels was in the range 97.22% (control)-68.44% (1 mM).

**Figure 4:**
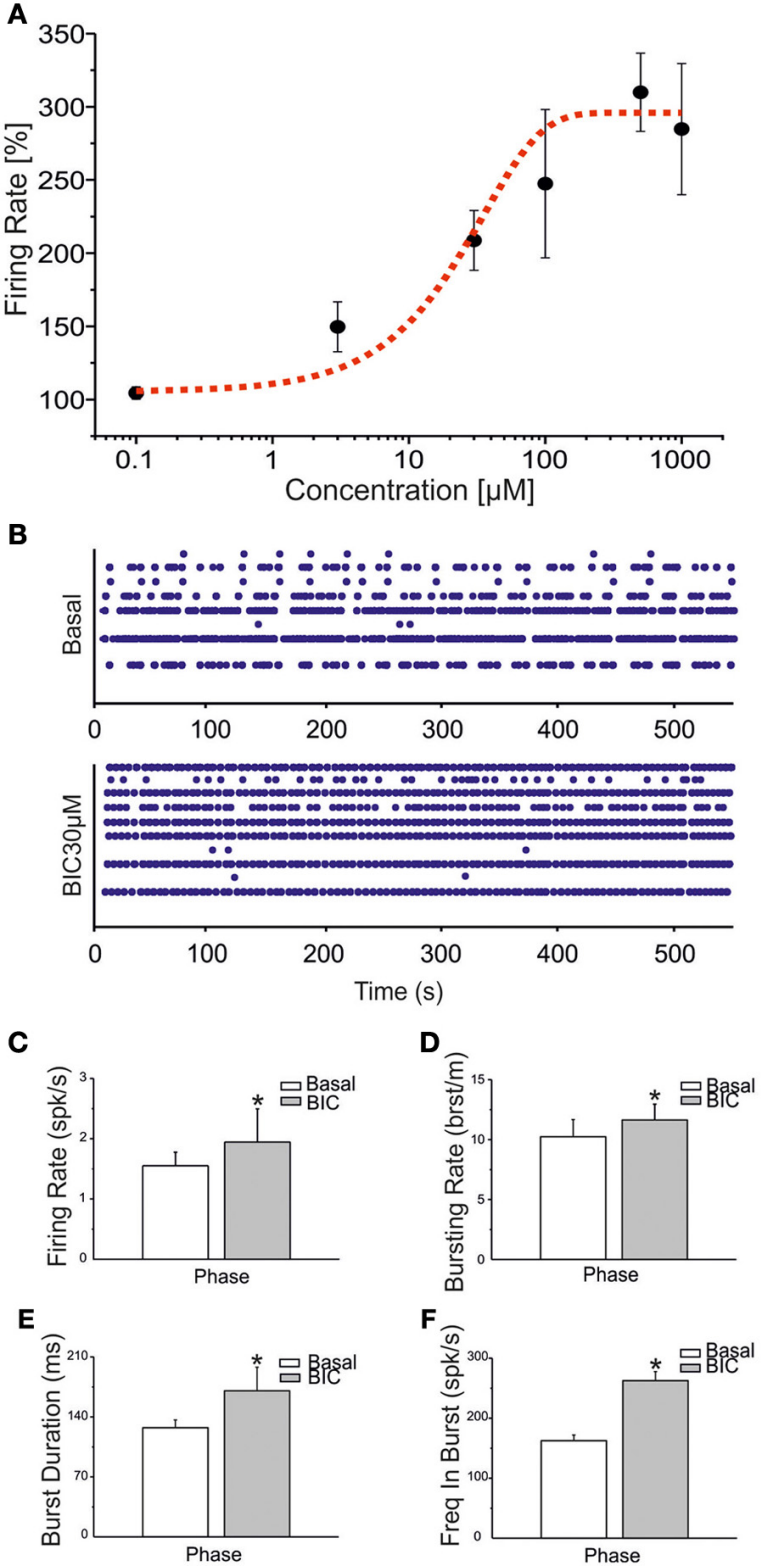
**BIC enhances bursting behavior in networks of hippocampal neurons. (A)** Dose-response curve for increasing concentrations of BIC (100 nM, 3 μ M, 30 μ M, 100 μ M, 500 μ M, 1 mM) was evaluated on a set of four experiments. The black line represents the mean response curve obtained by analyzing the changes in firing rate for the considered batch of experiments. The fitting of the dose-response curve by means of the Hill equation is the dotted red curve (*R*^2^ = 0.91). The EC50 value is reported in the text. **(B)** Raster plots showing 600 s of spontaneous (top) and pharmacologically induced (bottom) activity for a representative experiment. Drug concentration: 30 μM. **(C–F)** Bar graph for firing rate, bursting rate, burst duration and frequency intra burst during the basal phase (white bar) and during BIC treatment (gray bar). Statistics in **(C–F)** were performed using the Mann–Whitney *U*-test, ^*^p < 0.05.

In order to simulate ictal seizures in network of hippocampal neurons, we applied (BIC) at increasing concentrations to a set of four different cultures. BIC increased the firing rate in a concentration-dependent manner, as reported in Figure 4A. The effective concentration yielding a 50% response (EC50) was evaluated, according to the fitting of the concentration-response curve through the Hill equation (Frega et al., 2012). The computed EC50 was 29.75 ± 13.33 μM (mean ± SE). At 30 μM, BIC induced a significant increase in the network synchronization (Figure 4B), firing rate (Figure 4C), bursting rate (Figure 4D), burst duration (Figure 4E) and frequency in bursts (Figure 4F). In subsequent experiments, to evaluate the effects of CBZ and VPA on ictal-type activity avoiding ceiling or flooring effects, we pre-treated hippocampal cultures with 30 μM BIC.

**Figure 5:**
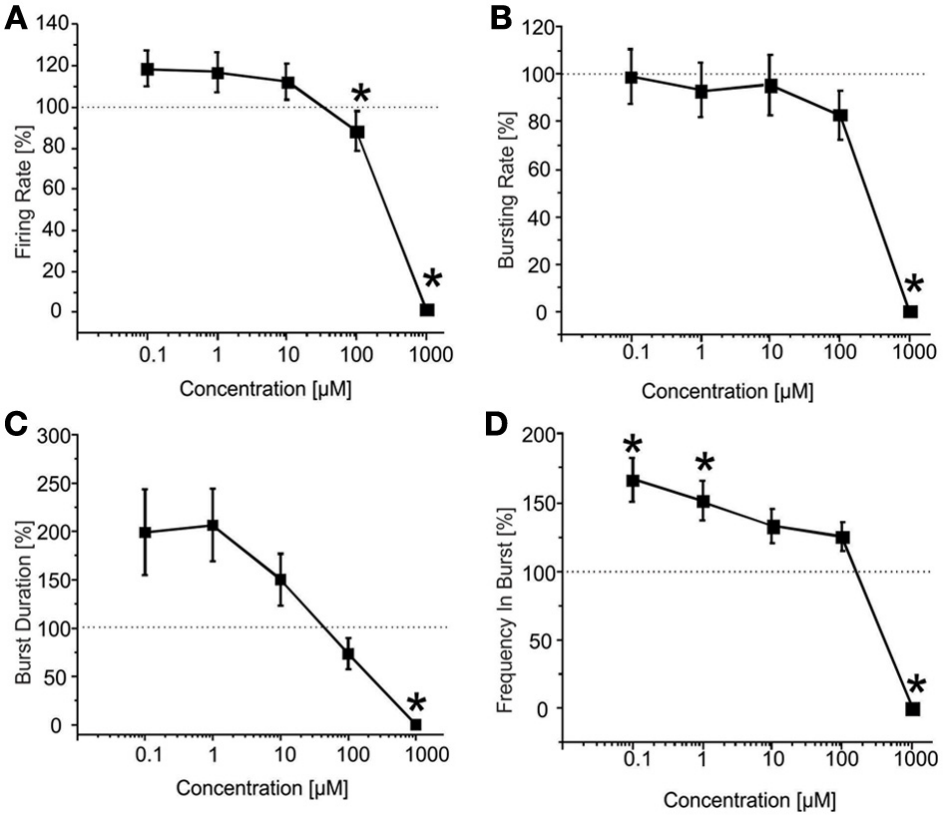
**Effects of increasing concentrations of CBZ upon BIC treatment (30 μM) on the firing and bursting dynamics of hippocampal networks. (A)** CBZ was applied to the culture medium at increasing concentrations (100 nM, 1 μ M, 10 μ M, 100 μ M, 1 mM), in presence of BIC (30 μ M). We found that in the analyzed group of experiments (*N* = 37; culture age 20.0 ± 0.6 DIV, MFR 1.3 ± 0.3 spikes/s, mean ± SE), the firing rate significantly decreased compared to the BIC condition for CBZ 100 μM and 1 mM. **(B)** The bursting rate followed a constant profile, showing no significantly change for CBZ 100 nM–100 μ M. Only at 1 mM CBZ there was a significant decrease of the considered parameter. **(C)** Burst duration decreased in a significant way only at CBZ 1 mM. **(D)** Mean frequency of spikes within the bursts (MFB) statistically increased compared to the control at CBZ 100 nM and 1 μ M, while it significantly decreased at CBZ 1 mM. Statistics in **(A–D)** were performed using One-Way ANOVA followed by Bonferroni's test, ^*^p < 0.05.

CBZ (*N* = 37 cultures) reduced the BIC-induced firing rate in a significant manner at high concentrations (i.e., 100 μM and 1 mM), as shown in the drug-response curve (Figure 5A). Bursting rate (Figure 5B) and burst duration (Figure 5C) followed a trend comparable to that of the firing rate, but the significant decrease was appreciated only at 1 mM. On the contrary, spike frequency intra burst (Figure 5D) showed a significant increase for low doses of the drug, and decreased abruptly for the highest concentration. For this experimental group, the percentage of active channels was in the range 98.11 and 97.22% (BIC alone and plus CBZ 100 nM) till 62% (CBZ 1 mM).

In the presence of BIC, VPA (*N* = 44 cultures) triggered dual behaviors in hippocampal networks. In one group of cultures, we observed a decrease of firing at low VPA concentrations, which return to BIC-evoked levels at high concentrations of VPA. In a second group of cultures, we observed a general concentration-independent decrease of firing. We therefore, analyzed the two groups separately.

**Figure 6:**
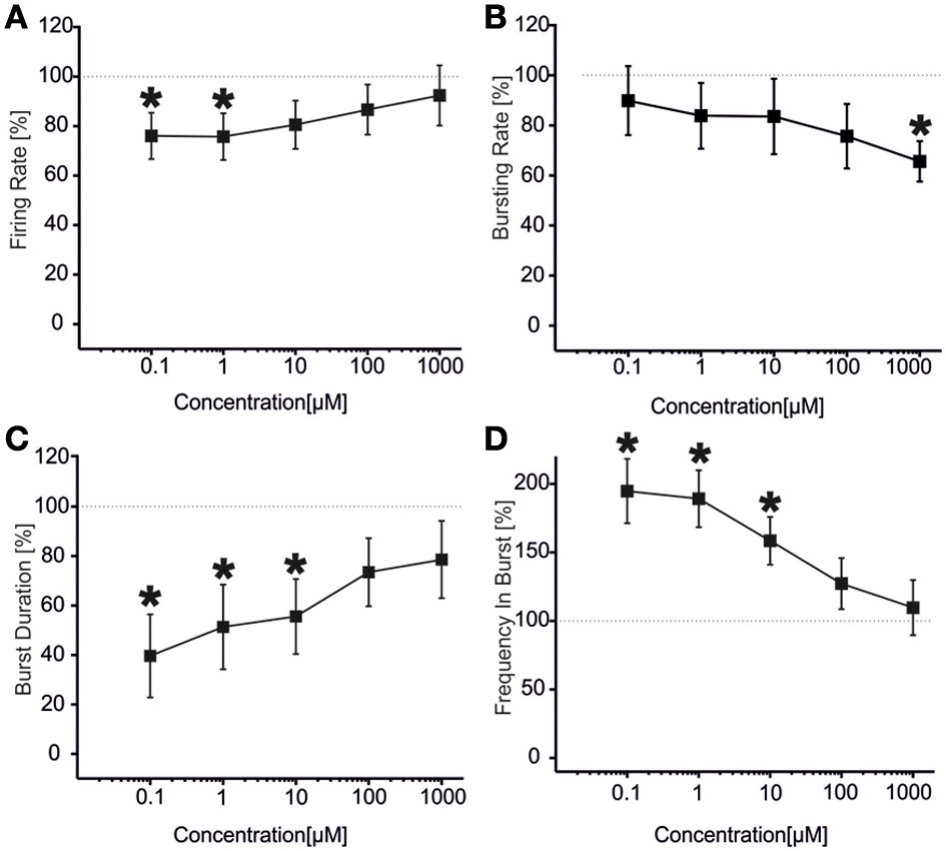
**Effects of increasing concentrations of VPA upon BIC treatment (30 μM) on the firing and bursting dynamic of hippocampal networks in a first group of experiments. (A)** VPA was applied in the culture medium at increasing concentrations (100 nM, 1 μ M, 10 μ M, 100 μ M, 1 mM), in presence of BIC (30 μ M). We found that in this group of experiments (*N* = 12; culture age 18.2 ± 0.2 DIV, MFR 1.4 ± 0.3 spikes/s, mean ± SE), the firing rate was significantly reduced at low doses of VPA (i.e., 100 nM–1 μ M), while it reached values comparable with the BIC condition for higher doses (10 μ M–1 mM). **(B)** The bursting rate significantly decreased compared to the BIC condition at 1 mM. **(C)** Burst duration had a significant decrease for VPA 100 nM till 10 μ M, while it reached values comparable with the BIC condition for higher concentrations (100 μ M–1 mM). **(D)** Mean frequency of spikes within the bursts statistically increased for the range of concentrations 100 nM–10 μ M, while it reached values comparable to the BIC phase for higher concentrations (i.e., 100 μ M–1 mM). Statistics in **(A–D)** were performed using One-Way ANOVA followed by Bonferroni's test, ^*^p < 0.05.

The results for the first group of experiments (*N* = 12 cultures) are shown in Figure 6. We can observe a significant decrease in the firing rate (Figure 6A) for low concentrations of VPA (i.e., 100 nM and 1 μM), accompanied by no change in the bursting rate (Figure 6B) for the same doses. At concentrations of 100 nM–10 μM, burst duration was significantly reduced (Figure 6C), while the frequency intra burst was almost doubled compared to culture treated with BIC alone (Figure 6D). For high concentrations of the drug (100 μM and 1 mM), the firing rate (Figure 6A), burst duration (Figure 6C) and frequency intra burst (Figure 6D) returned to values comparable to those achieved by BIC alone, while the bursting rate decreased significantly at 1 mM VPA (Figure 6B). In this group of experiments, the number of active channels remained high in all experimental conditions, starting from 99.11 and 96.55% (BIC alone and plus VPA 100 nM) to 96.55% (VPA 1 mM).

**Figure 7:**
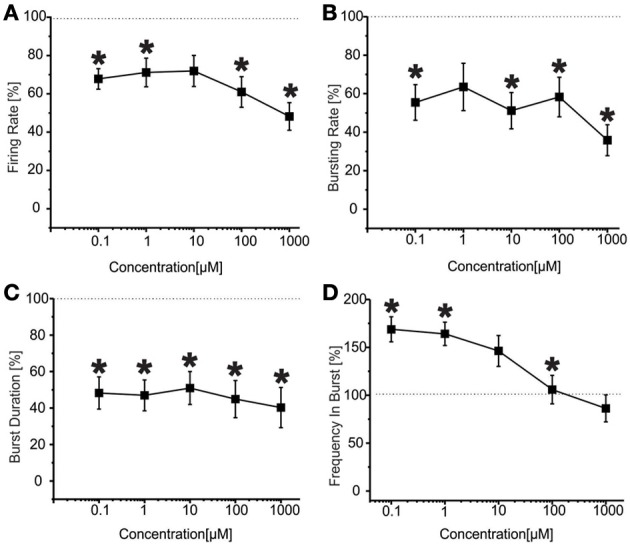
**Effects of increasing concentrations of VPA upon BIC treatment (30 μ M) on the firing and bursting dynamics of hippocampal networks in a second group of experiments. (A)** VPA was applied in the culture medium at increasing concentrations (100 nM, 1 μ M, 10 μ M, 100 μ M, 1 mM), in presence of BIC (30 μM We found that in the analyzed group of experiments (*N* = 32, culture age 21.8 ± 0.7 DIV; MFR 1.8 ± 0.3 spikes/s, mean ± SE), the firing rate significantly decreased compared to the control at low and high concentrations (i.e., 100 nM, 100 μ M, 1 mM), while it stayed the same for medium doses (1–10 μ M). **(B)** Bursting rate significantly decreased compared to the control for all the concentrations apart 1 μ M. **(C)** Burst duration significantly decreased for the entire range of tested concentrations. **(D)** Mean frequency of spikes within the bursts statistically increased for the range of concentrations 100 nM–1 μ M, while it reached values comparable to the control for higher concentrations (i.e., 10 μ M–1 mM). Statistics in **(A–D)** were performed using One-Way ANOVA followed by Bonferroni's test, ^*^p < 0.05.

The results for the second group of experiments (*N* = 32) are reported in Figure 7 From the analysis of the network parameters, we can note that VPA significantly reduced firing rate (Figure 7A), bursting rate (Figure 7B) and burst duration (Figure 7C) with similar effects at all concentrations tested. In fact, with respect to BIC alone, VPA decreased the firing rate by 35.9 ± 4.4%, the bursting rate by 47.2 ± 4.7% and the burst duration of about 53.7 ± 1.8% and the reduction was overall constant over the concentrations evaluated. The mean frequency intra burst (Figure 7D) was significantly increased at low concentrations (100 nM–1 uM) of VPA and returned to the levels of cultures treated with BIC alone at high concentrations. The percentage of active channels was 95.33% for BIC alone, 92.55% for VPA 100 nM and 70.33% for VPA 1 mM.

**Figure 8:**
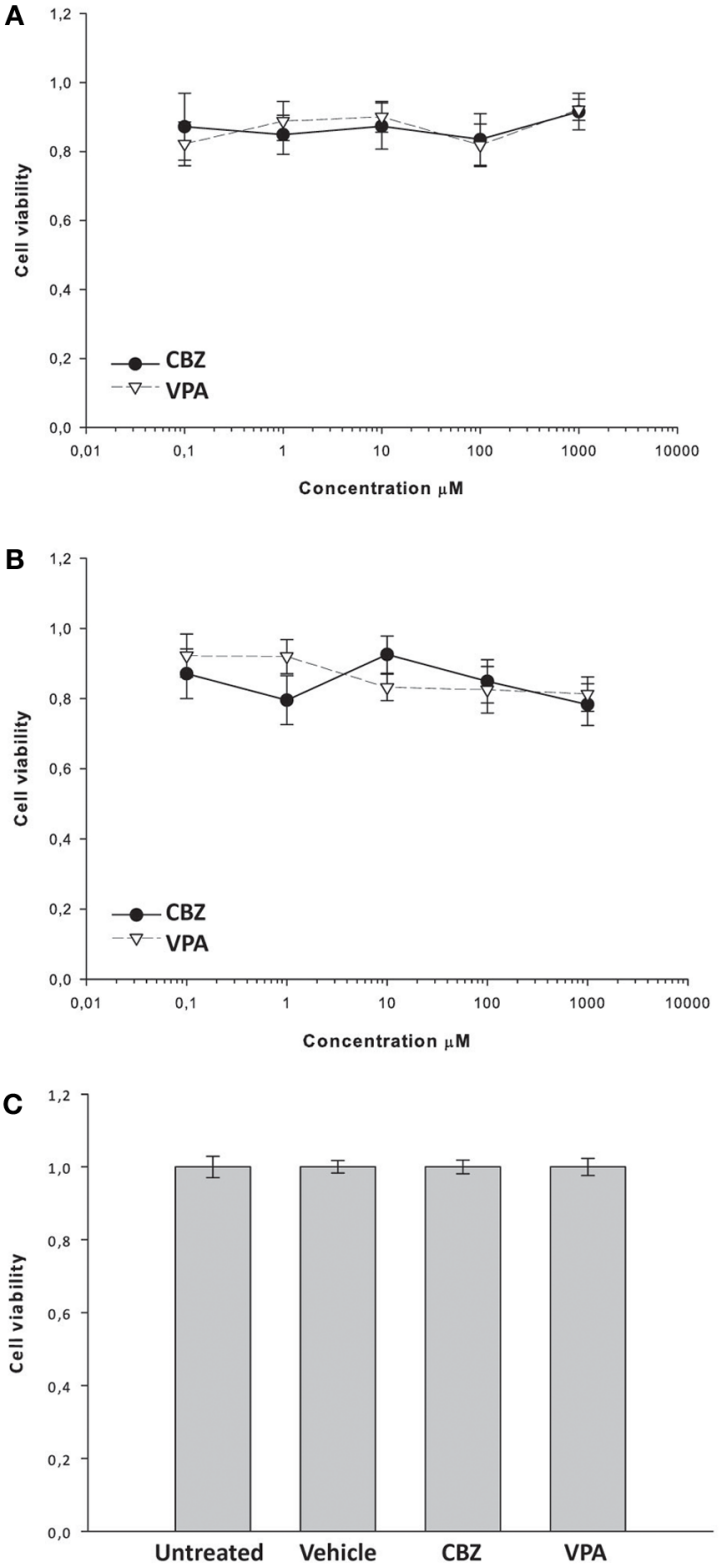
**Effects of CBZ and VPA on the viability of primary hippocampal neurons.** Hippocampal cultures were incubated for 1 h **(A)** or 5 h **(B)** in the absence or presence of CBZ (0,1–1000 μM), VPA (0,1–1000 μM) and/or vehicle. In a subset of cultures, increasing concentrations of CBZ or VPA were sequentially added to the each well using the same volumes and time schedule used for MEA experiments **(C)**. Cell viability was assessed by using the MTT assay as described in the Materials and methods section. Cell viability after drug treatment was expressed as percentage of the viability of untreated cells. Vehicle (DMSO) alone did not significantly alter cell viability **(C)**. Data represent the mean cell viability ± SE.

To investigate whether the effects of VPA and CBZ were due to neuronal toxicity or death, we evaluated the effects of the drugs on the viability of primary hippocampal neurons. We treated 18-day old primary hippocampal cultures for 1 or 5 h with increasing concentrations of CBZ or VPA and determined the viability of the cells by using the MTT assay. In a subset of cultures, to examine potential effects of mechanical or osmotic stress, increasing concentrations of CBZ or VPA were sequentially added to the same wells using the same volumes and time schedule used for MEA experiments. Neither CBZ nor VPA affected the viability of hippocampal neurons after treatment for 1 h (Figure 8A), 5 h (Figure 8B), or after sequential addition of increasing concentrations of drugs (Figure 8C), ruling out any toxicity of the drugs in these experimental conditions.

## Discussion

Our findings demonstrate that the anticonvulsant drugs, CBZ and VPA, significantly alter the spontaneous and BIC-evoked network activity of mature murine hippocampal neurons *in vitro*. Indeed we found that high concentrations of CBZ (100 μM–1 mM) significantly reduce both spontaneous and BIC-evoked neuronal firing. Conversely, VPA achieves different effects. In fact, high concentrations of VPA (100 μM–1 mM) reduce the spontaneous network activity in terms of firing rate, bursting rate and frequency intra burst. However, when applied in the presence of BIC, VPA displays dual effects depending on the batches of experimental cultures. In one group of cultures, low concentrations of VPA (100 nM–1 μM) significantly reduce BIC-evoked firing rate and burst duration, while increasing the frequency intra burst. Alternatively, in other cultures, the effects of VPA on firing rate, bursting rate and burst duration are independent from its concentration and reach maximal reduction already at the lowest drug concentration used.

With the exception of the frequency intra burst, the effects of CBZ on neuronal network activity are similar in the absence or presence of BIC and are significant at concentrations >100 μM. In particular the highest concentration of CBZ (1 mM) completely shuts down both firing rate and bursting rate in naïve cultures and in cultures exposed to BIC. This effect is not due to generalized toxicity of the drug, as demonstrated by the fact that neuronal viability is not altered by exposure to increasing concentrations of CBZ for up to 5 h. The effects of CBZ on frequency intra burst are opposite in the absence or presence of BIC. CBZ reduces the spontaneous frequency intra burst at 1 mM. However, CBZ concentrations <1 mM significantly enhance the frequency intra bursts evoked by BIC alone, possibly due to the effects of high CBZ concentrations on the firing threshold of neurons (Otoom and Alkadhi, 2000).

VPA alone depresses spontaneous activity only at high concentrations. Instead, BIC-treated cultures result more sensitive to VPA and changes of neuronal network activity are also detected at low concentrations of the drug (i.e., 100 nM–10 μM), which are close to the therapeutically relevant concentrations in humans (plasma concentration required for satisfactory control of seizures in man 181–301 μM (MacNamara, 1996), unbound free fraction in plasma 13.2 ± 10.2 μg/ml, equal to 91.7–165.3 μM (Gidal et al., 1995); 82.8–150.5 μM unbound free fraction in plasma corrected by brain-to-plasma ratio of 0.91 (Scism et al., 1997). CBZ shows effects on our cultures at concentrations (100 μM–1 mM) close to those clinically relevant (49.8–116.6 μM brain concentration calculated on the basis of plasma levels of 8.4–17.2 μg/ml after repeated doses (Eichelbaum et al., 1975) and brain/plasma ratio of 1.4–1.6 (Friis et al., 1978). These results provide a good indication there is a relationship between the sensitivity of our networks to a drug and the expected effect of that compound *in vivo*. Nonetheless, we are still far from the potential use of the MEA-based approach in the clinical practice (O'Shaughnessy and Pancrazio, 2007).

We also found that VPA displays a dual behavior on BIC-treated cultures, achieving its effects on firing in a concentration-independent manner in the majority (≈70%) of neuronal cultures or acting only at low concentrations (100 nM–1 μM) in the remaining ones. This stochastic behavior of VPA in different culture batches may be due to several factors. First, despite our culturing conditions are quite standardized and achieve reproducible networks, as demonstrated in previous studies (Novellino et al., 2011), we can not exclude that the relative proportion of excitatory and inhibitory neurons or their maturation state may slightly vary from one culture to another, favoring diverse electrophysiological behaviors. Indeed, the set of experiments of Figure 6 was performed on slightly younger cultures (18.2 ± 0.22 DIV) than those of Figure 7 (21.8 ± 0.7 DIV). This may account for the dual effects of VPA on BIC-evoked activity. However, the basal mean firing rate of cultures used for experiments of Figures 6, 7 was not statistically different (1.38 ± 0.36 spikes/s for the first group and 1.89 ± 0.31 spikes/s for the second group, Student's *t*-test *p* > 0.05), arguing against developmental differences. Moreover, we have evidence that the GABA-A receptor subunit switch in these cultures happens around 7–10 DIVs (unpublished observations), much earlier than the aging timeframe used in this study (3rd week of culture). Indeed, all our networks respond to BIC administration in the same way (i.e., by increasing the firing rate), demonstrating the inhibitory effect of GABA at the considered ages. Second, neuronal culture variability may also imply variable expression of sodium and calcium ion channels subtypes, which may potentially account for the different responses. Lastly, published data indicate that the effects of VPA may depend on the time of exposure to the drug, with concentrations >5 mM achieving pro-convulsants effects on veratridine-induced epileptiform activity in rat hippocampal slices after prolonged exposure (Otoom and Alkadhi, 1999). However, this hypothesis is not supported by our observations, as we did not detect any time-dependent change within each 50 min experimental recording session at any given concentration in the absence or presence of BIC.

In conclusion, our results propose that MEA-coupled hippocampal cultures represent an interesting model system to investigate the acute effects of common antiepileptic drugs on the electrophysiological activity of neuronal networks *in vitro*. Such system could be worthwhile for investigating the long-term exposure to anticonvulsant compounds in view of developing novel neuropharmacological/neurotoxicological assays for drug screening. Nevertheless, our final considerations also provide an important indication for the necessity of international standardization and evaluation procedures for the MEA-based approaches (Johnstone et al., 2010), in order to possibly limit variability of the results.

### Conflict of interest statement

The authors declare that the research was conducted in the absence of any commercial or financial relationships that could be construed as a potential conflict of interest.
